# Percutaneous Balloon Pulmonary Valvuloplasty of Critical Pulmonary Stenosis and severe pulmonary stenosis in Neonates and Early Infancy: A Challenge in the Cyanotic

**DOI:** 10.34172/jcvtr.2021.33

**Published:** 2021-05-20

**Authors:** Hojjat Mortezaeian, Mohammadrafie khorgami, Negar Omidi, Yasaman khalili, Maryam Moradian, Raheleh Zamani, Esfandyar Nazari

**Affiliations:** ^1^Rajaie Cardiovascular Medical and Research Center, Iran University of medical sciences, Tehran, Iran; ^2^Cardiac Primary Prevention Research Center, Tehran Heart Center and Department of Cardiology, School of Medicine, Tehran University of Medical Sciences, Tehran, Iran

**Keywords:** Cyanotic Heart Disease, Pulmonary Valve Stenosis, Balloon Pulmonary Valvuloplasty

## Abstract

***Introduction:*** Pulmonary stenosis with an intact ventricular septum (PS-IVS) is one of the common causes of cyanotic heart disease in neonates with diverse morphologies as well as management and treatment protocols. The aim of this study was to evaluate short and midterm results of balloon pulmonary valvuloplasty (BPV) for this disorder.

***Methods:*** Between 2012 and 2016, Totally 45 neonates and infants under 6 months old were evaluated.The patients had a minimum right-to-left ventricular pressure ratio of 1, right-to-left shunting at the patent foramen ovale or atrial septal defect level, and tricuspid valve Z-scores higher than -4.

***Results:*** Immediately after the procedure, the right ventricular pressure dropped to the normal values in 8 (20%) patients. The immediate procedural success rate was seen in 42 (93.3%) cases: the right-to-left ventricular pressure ratio dropped to below 50% or the level of O2 saturation rose above 75%. Of three cases unresponsive to BPV, two of them underwent patent ductus arteriosus (PDA) stenting and one procedural death occurred. At 6 months’ follow-up, of 42 patients, this pressure was still with in the normal range in 36 (80%) infants, while it had returned to high values in 9 (20%) patients and necessitated repeat valvuloplasty. After BPV, severe pulmonary valve regurgitation was observed in14.2% patients; the condition was more common when high-profile noncompliant balloons were used.

***Conclusion:*** Balloon pulmonary valvuloplasty in infants with PS-IVS confers acceptable results insofar as it improves echocardiographic parameters and hemodynamic changes at short- and midterm followups.Balloon selection with sizes more than 1.2 of the diameter of the pulmonary valve annulus and the use of noncompliant high-pressure balloons results in higher degrees of pulmonary regurgitation.

## Introduction

Pulmonary stenosis with intact ventricular septum (PS-IVS) is one of the frequent causes of cyanotic heart disease in neonates and infants,^1^ requiring prompt management. Also, it is not a unique term because the diverse morphologies of this disorder call for different management approaches and its exact diagnosis requires the evaluation of clinical information, echocardiographic findings, and catheterization data.^2^

The size of the right ventricular (RV) cavity, size of the infundibulum of the right ventricular outflow tract (RVOT), and diameter of the tricuspid valve (TV) are important landmarks. Other variables with major roles in this regard include the right-to-left ventricular (RV/LV) pressure ratio, ductal prostaglandin dependency, severity of the clinical manifestations, and associated anomalies.

Until recently, surgical pulmonary valvotomy aimed at increasing the pulmonary blood flow was the treatment of choice; nevertheless, transcatheter balloon pulmonary valvuloplasty (BPV) is now the standard therapy for this disorder.^3,4^

The decompression of the RV with BPV with or without ductal stenting has been accompanied by various short- and long-term prognoses in different studies.^5-7^ Accordingly, we sought to evaluate both the short- and midterm results of Balloon pulmonary valvuloplasty in 45 neonates and infants with PS-IVS and the effects of our interventional procedures on the patients’ outcome.

## Materials and Methods

This study was a prospective review that 45 patients less than 6 months old admitted to our Center with a diagnosis of PS-IVS between May 2012 and July 2016 were evaluated. This study was approved by university ethical committee according to the Helsinki Declaration of the World Medical Association (Ethic code number:IR.RHC.REC.1391.70). Informed consent was obtained from the parents. Prostaglandin E1 (PGE1) was initiated if the level of O2 saturation was below 75%.

The inclusion criteria comprised age under six 6 months old, severe PS, RV/LV pressure ratio equal to or higher than 1, right-to-left shunting at the patent foramen ovale (PFO) or atrial septal defect level (ASD), and tricuspid valve Z-score greater than -4. The exclusion criteria consisted of stenosis of the supra-pulmonary valve and the peripheral pulmonary branches, association with other structural heart diseases, tricuspid valve Z-score less than -4, hypoplastic right ventricle, and right ventricle- dependent coronary perfusion.

The echocardiographic examinations were performed by a single operator using a Vivid 7 system (Vingmed 7; GE Medical System, Inc, Horten, Norway). The pulmonary valve was considered dysplastic when the valve was thick and had low motion with a small annulus size without post-stenotic dilation in the main pulmonary artery (PA). The size of the RV and the normality of the 3 segments of the right ventricle chamber (i.e., inlet, apex, and outlet) were assessed. In addition, the diameter of the tricuspid valve annulus was measured in the 4-chamber view and the tricuspid valve Z-score was calculated according to the patients’ body surface area and appropriate tables.^8^

Six months after pulmonary valvuloplasty, RV/LV pressure ratio was calculated by echocardiography. The pressure gradient across the pulmonary valve was measured by considering the pulmonary artery pressure normal in this group of patients, and the RV pressure was determined. In the presence of tricuspid valve regurgitation, the RV pressure was measured based on Bernoulli equation. Furthermore, the systolic aortic pressure was measured for the assessment of the left ventricle systolic pressure with an appropriate pressure cuff based on age and RV/LV pressure ratio was estimated.^9,10^

Contrast echocardiography was performed for a better evaluation of intra cardiac shunts, especially in the ASD level. The width of the regurgitation jet determined the severity of the tricuspid regurgitation as mild, moderate, or severe.

The right ventricle size and function, severity of tricuspid and pulmonary regurgitation (PR), and intra-atrial shunts in the ASD and patent ductus arteriosus (PDA) levels were evaluated post BPV and subsequently 1 and 6 months afterward in the outpatient follow-up.

Anesthesia was induced in under the observation of a cardiac anesthesiologist. The drug protocol consisted of intravenous midazolam and ketamine. Tracheal intubation was performed according to the patient’s condition. Oxygen saturation, arterial blood O_2_ level, and blood pressure were continuously monitored during the procedure. The intracardiac pressure was measured with a fluid-filled catheter system. Before and after BPV, the RV/LV pressure ratio and the right ventricle to pulmonary artery gradient were measured. A 4-F or 5-F Judkins coronary catheter was placed in the RVOT, and a 0.014-in guide wire was passed through the pulmonary valve into the ductus arteriosus and the descending aorta, if accessible, or the pulmonary branches.

Based on the severity of pulmonary stenosis, the balloon-to-pulmonary valve annulus diameter was calculated and a proper balloon valvuloplasty catheter was selected. In some patients, balloon dilation was done with smaller balloon sizes and gradually larger balloons were selected until the maximal diameter was used.

**Figure 1:**
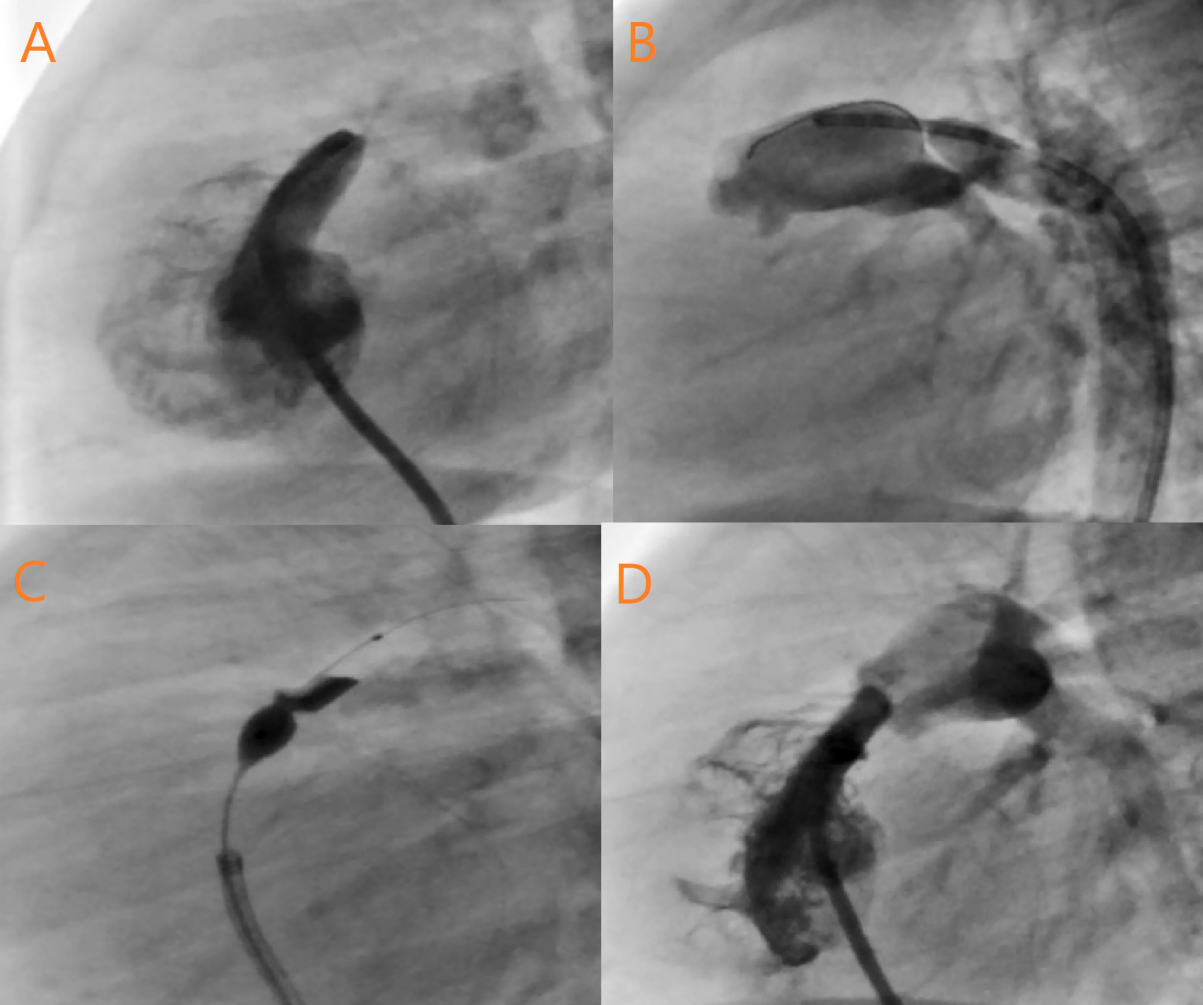
**A:** RV injection in Lateral view revealed Critical Valvar PS, Sever RVH,Small size RV,Elongated RVOT, Pin hole Fenestrated Pulmonary Valve, Doming and Thickening of Cusps.

The BPV procedure was considered successful if the guide wire and the intended pulmonary balloon were successfully passed through the pulmonary valve and an appropriate waist was formed (Figure 1).

**B:** DAO injection in Lateral View showed Tubular and long shape PDA, Significant Left to Right Shunt,PA filling via PDA. **C:** Ante grade wiring of PA by CTO wire, PTPC by Mini- Tyshak Balloon size 7-20 mm. **D:** Final injection in RV at Lateral View showed opening of Pulmonary Valve, Patent and good ante grade Flow.

The selection of the appropriate balloon size was based on the echocardiography and catheterization measurements. The diameter of the pulmonary valve annulus was measured in the short-axis view via echocardiography. During the cardiac catheterization, the diameter of the pulmonary annulus was calculated in the anteroposterior and lateral views after the injection of the contrast medium into the RV.

Also, we select the balloon type based on several factors including: the age and weight, ability of passing venous introducer sheaths, pulmonary valve cusp thickness, and limitation of pulmonary valves motion.

If the RV pressure after balloon pulmonary valvuloplasty dropped to less than 36 mm Hg, it was considered a normal RV pressure.

The procedure was deemed successful if the RV/LV pressure gradient was decreased to below 50% of the left ventricle pressure or if the level of O_2_ saturation was increased to above 75% and Prostaglandin E1 was discontinued.

### Statistical analysis

The continuous data with normal and non-normal distributions were reported with means and standard deviations, respectively, and the categorical data were reported with frequencies. The Kruskal–Wallis test, Pearson χ^2^ test, and Friedman test were applied for the statistical analyses. SPSS (IBM SPSS Statistics for Windows, version 22.0, Armonk, NY: IBM Corp) was used for all the analyses.

## Results

Forty-five patients, consisting of 23 (51.1%) males and 22 (48.9%) females, at a mean body weight of 4.8(5.2 ± 1.8 (kg were evaluated between May 2012 and July 2016. The demographic and data of Echocardiography and catheterization depicted in Table 1 and Table 2 respectively. The types of balloons used for pulmonary valve valvuloplasty and degree of pulmonary valve regurgitation showed in Table 3.Dysplastic pulmonary valves were reported in 3 patients and PDAs with left-to-right shunts and the supply of the pulmonary blood flow from the patent ductus arteriosus were observed in 23 (51.1%). Prostaglandin E1 was initiated in 4 (8.8%) patients because they had complete PDA dependent pulmonary blood circulations and severe cyanosis.

**Table 1:** Demographic, clinical and catheterization data of the patients with critical PS-IVS.

**Variables (%)**	**N**
Age	
< 3 month	22(51.1)
3–6 month	23(49.9)
Gender	
Female	22(48.9)
Male	23(51.1)
Weight*	4.8 (1.8)
General anesthesia and tracheal intubation	38 (84.4)
O2 saturation#	71%
ASD in patients	42(93.3)
PDA (with left to right shunt)	23(51.1)
PGE1 dependency	4(8.9)
ASD (with right-to-left shunting)	45(100)
PDA stenting	2 (4.4)
Balloon size/PV annulus	
<100%	1 (2.3)
100–120%	36 (80)
120–150%	8 (17.7)
BPV without Balloon predilation	32(71.1)
BPV with Balloon predilation	13(28.8)

Data expressed as number/percent except *as Kg (mean/SD ) and # as mean

**Table 2:** Echocardiographic and catheterization data in the patients with critical PS-IVS before and 6 months after BPV.

**Parameters**	**before BPV**	**after BPV**
TV regurgitation		
Mild	25 (56.6)	32 (71)
Moderate	6 (12.3)	12 (26.6)
Severe	14 (31.1)	1 (2.2)
PV regurgitation		
Mild		32 (71)
Moderate		7 (15.5)
Severe		6 (14.2)
-TV Z-score		
> -2	28 (62.2)	
-2 to -4	9 (20)	
> 0	8 (17.8)	
Response to Therapy		
Immediately after BPV		42 (93)
After 6 months		36 (80)
Normal RV pressure (<36 mm Hg)		
Immediately after BPV		9 (20)
After 6 months		36 (80)

Data expressed as number/percent,BPV: Balloon pulmonary valvuloplasty

**Table 3:** The types of conventional balloons used for pulmonary valve valvuloplasty and degree of pulmonary valve regurgitation (PR) occurred with each type.

**Type of balloon and patients number**	**Mild**	**Moderate Severe**	
Minityshake (28)	24	2	2
Tyshake (5)	5	0	0
Sterling (3)	2	1	0
Z-Med 2,Z-Med x (3)	0	2	1
Evercross (4)	1	1	2
Opta pro (2)	0	1	1

Data expressed as number

### Electrocardiographic (ECG) findings

At the commencement of the study, all the patients had normal sinus rhythms on surface ECG. In most of them (77.7%), right frontal axis deviation was observed. Eight (17.7%) patients had normal axes and only 2 (4.4%) had superior axes. Patterns of right ventricle hypertrophy were observed in the ECG of the majority of the study population (93.3%), and 3 patients had left ventricle hypertrophy.

***Echocardiography finding*** *s*

All the 45 patients had ASD or PFO. The tricuspid valve regurgitation was severe in 31.1% and moderate in 12.3% of the study patients, with the rest having mild TR. The RV size was normal (tricuspid valve Z-score > -2) in 28 (62.2%) patients, small (tricuspid valve Z-score = -2 to -4) in 9 (20%), and larger than normal in 8 (17.8%). After the procedure, 32 (71.1%) patients had trivial-to-mild tricuspid valve regurgitation and only 1 patient had severe tricuspid valve regurgitation. The change in the severity of tricuspid valve regurgitation was significant after BPV (*P* = 0.034).

In the post- Balloon pulmonary valvuloplasty echocardiography, pulmonary regurgitation was severe in 6 (14.2%) infants, moderate in 7 (15.5%), and mild in 32 (71.1%).

### Procedures

BPV procedure was performed successfully in all the 45 cases. The level of O_2_ saturation was increased to above 75% in 42 patients.The pulmonary balloon size was between 100% and- 120% of the diameter of the pulmonary valve annulus in 36 (80%) patients and between 120% and 150% in 8 (17.7%). In 1 (2.2%) infant, the balloon size was smaller than the diameter of the pulmonary valve annulus because a larger balloon failed to pass through the pulmonary valve following coronary balloon predilation. Predilation of the balloon for the pulmonary valve was not required in 32 (71.1%) cases, while predilation was done in 13 (28.8%) with smaller to larger balloons sequentially in 2 to 3 steps with 0.014-in coronary guide wires. This procedure was repeated once more due to significant residual pulmonary stenosis after the first pulmonary valvuloplasty in 8 cases.

Of three cases that did not respond to initiate BPV, PDA stenting was done in 2 infants with Genesis Blue 4.5 Xorms stents. After PDA stenting, O_2_ saturation was increased, and the infants were extubated. During follow up, in first patient stent of PDA was occluded spontaneously and PTPC repeat again 1 year later and second patient refer to One and half repair surgery and PDA stent was occluded during surgery.

The RV was equal with the LV pressure in 23 (48.8%) cases and suprasystemic in 23 (51.1%). The RV dropped to less than 36 mm Hg immediately after the procedure in the catheterization laboratory in 8 (20%) infants. The immediate success rate was seen in 42 (93.3%) patients (the RV/LV pressure ratio dropped to < 50% or O_2_ saturation increased to > 75%). At 6 months’ follow-up, the right-to-left ventricular pressure ratio was still within the normal range in 36 (80%) infants, but it had returned to high values in 9 (20%) and required repeat valvuloplasty. Of these 9 patients, three infants did not respond to repeat BPV and were, therefore, subjected to surgical valvotomy but six patients respond to BPV appropriately. During the follow-up, the change in the pressure was significant after 6 months (*P* = 0.009). Thirteen (28.8%) patients were treated with beta-blockers post procedurally and after 6 months, the RV pressure was normal in this group.

The PDA remained patent 6 months after the valvuloplasty in 4 patients and it was closed via an interventional procedure, while the PDA closed spontaneously in the other patients. The ASD or PFO closed spontaneously in 33 (73.3%) infants.

### Complications

Trauma to the vascular access was observed in 7 patients, all of whom were under 3 months old. In the presence of lower-limb pallor, coldness, and pulselessness, intravenous heparin was infused and 3 patients needed thrombolytic therapy.

There was 1 death in a neonate less than 1 month old due to procedural complications. This patient suffered the rupture of the RVOT during catheterization resulting in tamponade and underwent pericardiocentesis and urgent surgical pericardiotomy but expired 5 days later. The hospitalization period was less than 3 days in 3 (6.6%) patients, 3 to 5 days in 20 (44.4%), 5 to 7 days in 14 (31.1%), and more than 7 days in 8 (17.7%).

## Discussion

Although in older studies the number of pulmonary balloon dilations and the immediate success rate were important, recent studies attach more significance to the mid- and long-term outcomes of the procedure and focus on several factors that may affect the outcome. In the present study, we comprehensively evaluated the outcome of the Balloon pulmonary valvuloplasty procedure in infants less than 6 months old with a 6-month follow-up.

Immediate procedural success rate was 93% in our study. The overall success rate in different studies varies between 85% to 87%. ^11-13^ Coronary balloon pre-dilation required in 29% of patients which was in line with Hassan et al (30%) but in Yucel et al study this rate was 18%. ^13,14^

After BPV by immediate procedural success rate, we observed a significant reduction in the RV pressure (<36 mm Hg) in a small number of our patients (20%), which is consistent with other studies.^15-17^ Nonetheless, at 6 months’ follow-up, the RV had returned to the normal values in most of our patients (80%).

Humpl et al,^17^ reported that after BPV on 30 patients, the mean RV was 42 mm Hg in 9 patients and 61 mm Hg in the remainder, who needed intervention.

In our study, about one-third of cases has severe TR before procedure, nevertheless, at six month follow up, most (71%) had mild TR and only one patients had severe TR. Yucel et al,^14^ observed moderate and severe TR in significant percentage of total 55 neonate respectively. The authors reported that the need for the augmentation of the blood flow after BPV was more in the patients with severe TR. Elsewhere, Saad et al,^11^ studied 76 infants with severe PS and reported a reduction in the incidence of TR from 56% to 20% at 6 months’ follow-up following BPV.

On echocardiography, larger RV is often accompanied by RV systolic dysfunction and severe TR. In cases with small right ventricle sizes (Z-score = -2 to -4) on echocardiography, the right ventricle cavity is severely hypertrophied but the systolic function is nearly normal. In this group of patients, right-to-left shunting via the interatrial communication and cyanosis are frequent.

In the current study, the majority of the patients with persistently high RV pressures at 6 months’ follow-up (20%) needed repeat valvuloplasty. Three cases failed to respond to valvuloplasty and underwent surgery. This condition occurred in a group with small, dysplastic, and thick pulmonary valves and severely hypertrophied high right ventricle chambers.

In our study, most patients were hospitalized for between 3 and 7 days, but there was no significant correlation between age and the mean weight and the length of hospitalization. The median length of hospital stay was 11.6 days in a study by Odemis et al,^18^ on 15 neonates.

In our study, we observed trauma to the vascular access in 7 patients, all of whom were under 3 months old. This rate chimes in with those reported by Chubb et al,^15^ and Humpl et al,^17^. In study of Hoetama et al, BPV was done on 8 neonates with transjugular approach and they mentioned that using BPV with transjugular access is safe in neonate with critical PS.^19^

We had 1 death (in 45 patients) due to RVOT rupture and tamponade. In the study by Chubb et al,^15^ on 39 neonates with Critical pulmonary stenosis or atresia with intact ventricular septum, the 30-day mortality after BPV was 6 patients, 2 of them died whom expired due to tamponade. Humpl et al ^17^ reported 5 postprocedural deaths (3 early and 2 late).

In our study, the use of low-profile semicompliant balloons in most of the patients was correlated with mild post-procedural pulmonary regurgitation, whereas the use of high-profile noncompliant balloons was correlated with a higher incidence rate of moderate and severe PR. Moreover, when the balloon size was between 120% and 150% of the diameter of the pulmonary valve annulus, there was a high degree of PR in comparison with when balloon sizes less than 120% were selected. Therefore, in our study, the factors with an impact on pulmonary regurgitation were balloon selection with sizes more than 1.2 the diameter of the pulmonary valve annulus, use of noncompliant high-pressure balloons, and larger right ventricle chambers and annuli. It seems that the prevention of severe PR requires not only the previous balloon-selection methods such as the diameter of the pulmonary valve annulus but also more accurate methods such as balloon selection based on the patient’s body surface area.

factors that increase the probability of repeat BPV or surgery—including dysplastic pulmonary valves, pulmonary valve diameter, TV diameter, RV size (especially in cases with TV Z-scores between (-2 and -4), balloon size, and type and profile of the balloon.

## Conclusion

The BPV procedure is now deemed conventional therapy for neonates and infants with PS-IVS. The treatment protocol, however, should be on a patient-by-patient basis because there are a number of factors—including the RV morphology, patient’s general condition, and issues related to the interventional procedure. In our study, the right ventricle indices were abnormal immediately after BPV, but our midterm follow-up demonstrated that the indices had returned to the normal values. Another salient point is that favorable results are dependent on the selection of proper balloons. Undoubtedly, further studies with longer follow-up durations are required to shed sufficient light on the other aspects of the management protocol.

## Acknowledgments

we thank the staff and participants in Rajaie Cardiovascular, Medical, and Research Center in pediatric field.

## Competing Interest

None.

## Ethical Approval

The study protocol was approved by the Ethics Committee and the Review Board of Iran University of Medical Sciences (IR.RHC.REC.1391.70)

## Funding

we have not any financial support from person, industry and organs.
